# Comparison of metal versus plastic stent for preoperative biliary drainage in patients with pancreatic cancer undergoing neoadjuvant therapy: a meta-analysis and systematic review

**DOI:** 10.1186/s12876-023-02874-5

**Published:** 2023-07-12

**Authors:** Yunxiao Lyu, Shenjian Ye, Bin Wang

**Affiliations:** Department of Hepatobiliary Surgery, Dongyang People’s Hospital, 60 West Wuning Road, Dongyang, 322100 Zhejiang China

**Keywords:** Pancreatic cancer, Neoadjuvant therapy, Metal stent, Plastic stent, Meta-analysis, Systematic review

## Abstract

**Background:**

This study was performed to compare a metal stent (MS) and plastic stent (PS) in terms of efficacy and complications during neoadjuvant therapy (NAT) and the perioperative period.

**Methods:**

We performed an electronic search of the following databases until 1 June 2022: PubMed, Embase, Web of Science, Cochrane Central Register of Controlled Trials, and ClinicalTrials.gov. Studies comparing an MS versus PS for PBD in patients with pancreatic cancer undergoing NAT were included.

**Results:**

The meta-analysis showed that use of an MS was associated with lower rates of reintervention (*p* < 0.00001), delay of NAT (*p* = 0.007), recurrent biliary obstruction (RBO) (*p* = 0.003), and cholangitis (*p* = 0.03). There were no significant differences between the two groups in terms of stent migration (*p* = 0.31), postoperative complications (*p* = 0.20), leakage (*p* = 0.90), and R0 resection (*p* = 0.50).

**Conclusions:**

Use of an MS for PBD in patients with pancreatic cancer undergoing NAT followed by surgery was associated with lower rates of reintervention, delay of NAT, RBO, and cholangitis compared with use of a PS. However, the postoperative outcomes were comparable between the MS and PS. Further studies on this topic are recommended.

**Supplementary Information:**

The online version contains supplementary material available at 10.1186/s12876-023-02874-5.

## Background

Radical surgery is a curative treatment for pancreatic head cancer. However, only about 15–20% of patients are potential candidates for resection at the diagnostic stage [1]. With the development of chemotherapy and radiotherapy, neoadjuvant therapy (NAT) [including neoadjuvant chemotherapy (NAC) or neoadjuvant chemoradiotherapy (NACRT)] has drawn attention regarding its application in resectable and borderline resectable pancreatic cancer because of its ability to reduce the tumor size and increase the R0 resection rate [2–4]. However, obstructive jaundice is one of the primary symptoms of pancreatic head cancer, and NAT in such cases would be time-consuming because of the need to maintain biliary drainage. Endoscopic biliary drainage is considered superior to percutaneous biliary drainage in terms of peritoneal dissemination and patient comfort [5]. A metal stent (MS) and plastic stent (PS) are two types of stents commonly used for preoperative biliary drainage (PBD) in patients undergoing endoscopic biliary drainage. An MS appears to be the optimal choice for unresectable pancreatic cancer [6]. Compared with a PS, an MS has the advantages of longer patency and a lower reintervention rate. Nevertheless, although several studies have compared the efficacy and safety of an MS versus PS for NAT in patients with resectable or borderline resectable pancreatic cancer, there is no standard viewpoint on the best type of stent [7, 8]. The preoperative and postoperative outcomes between an MS and PS remain controversial. This study was performed to compare an MS and PS for PBD in patients with pancreatic cancer undergoing NAT.

## Materials and methods

This systematic review and meta-analysis were reported in line with the Preferred Reporting Items for Systematic Reviews and Meta-Analyses (PRISMA) guidelines [9].

### Search strategy

Two authors independently conducted a thorough electronic search of the PubMed, Embase, Web of Science, Cochrane Central Register of Controlled Trials (CENTRAL), and ClinicalTrials.gov databases until 1 June 2022 to identify studies comparing an MS and PS for PBD in patients with pancreatic cancer undergoing NAT. English-language search terms included but were not limited to the following: “ERCP,” “endoscopic,” “stent,” “metal,” “endoscopic retrograde cholangiopancreatography,” “plastic,” “neoadjuvant,” “chemoradiation,” “neoadjuvant chemoradiation therapy,” and “pancreatic cancer.” The search was restricted to human subjects and English-language articles. The references of the articles identified after the initial search were also manually reviewed. Any discrepancy in article selection was resolved by consensus.

### Inclusion and exclusion criteria

Studies that met the following criteria were included in the meta-analysis. (1) The study compared any MS and PS for PBD in patients with pancreatic cancer undergoing NAT. (2) Information was provided on reintervention, delay of NAT, recurrent biliary obstruction (RBO), cholangitis, migration, R0 resection, and postoperative complications. (3) The original article was published in English. Abstracts were included as long as they met the inclusion criteria and provided the data needed for the analysis. We excluded (1) studies that did not provide sufficient data and (2) case series, non-comparison studies, and non-human studies.

### Outcome measures and data extraction

The outcome measures were reintervention, delay of NAT, RBO, cholangitis, migration, R0 resection, and postoperative complications. Reintervention was defined as endoscopic biliary drainage necessitated by the appearance of elevated hepatobiliary enzyme and total bilirubin levels and/or concomitant cholangitis. RBO is defined as a re-elevation of total bilirubin. R0 resection was defined as no microscopic or macroscopic tumor. The definition of operative complications adopts the definition of postoperative complications in the original study. We abstracted the data of interest from the included studies onto a standardized form, including the author, year of publication, type of study, country in which the study took place, sample size, patient age, and pancreatic cancer status. Conflicts in data abstraction were resolved by consensus and by referring to the original article. EndNote X8 (Thomson Reuters, Toronto, Ontario, Canada) was used to remove duplicate studies.

### Quality assessment

The quality of the included nonrandomized studies was assessed in accordance with the Newcastle–Ottawa scale [10]. The scoring system included the following criteria: random sequence generation, allocation concealment, blinding of participants and personnel, blinding of the results assessment, incomplete results data, selective reporting, and other sources of bias. The Cochrane collaboration tool was used to assess the quality of the randomized controlled trials (RCTs) by evaluating methods of randomization and allocation concealment, performance, and detection of bias [11].

### Statistical analysis

All statistical analyses were performed using Review Manager (RevMan) version 5.3 software (The Nordic Cochrane Centre, The Cochrane Collaboration, Copenhagen, Denmark). Odds ratios (ORs) with 95% confidence intervals (CIs) were used for dichotomous outcomes. Publication bias was evaluated by the χ^2^ test and funnel plots. Heterogeneity among studies was evaluated by the χ^2^ test. A two-tailed *p* value of < 0.05 was considered statistically significant.

## Results

### Study selection and trial characteristics

**Fig. 1:**
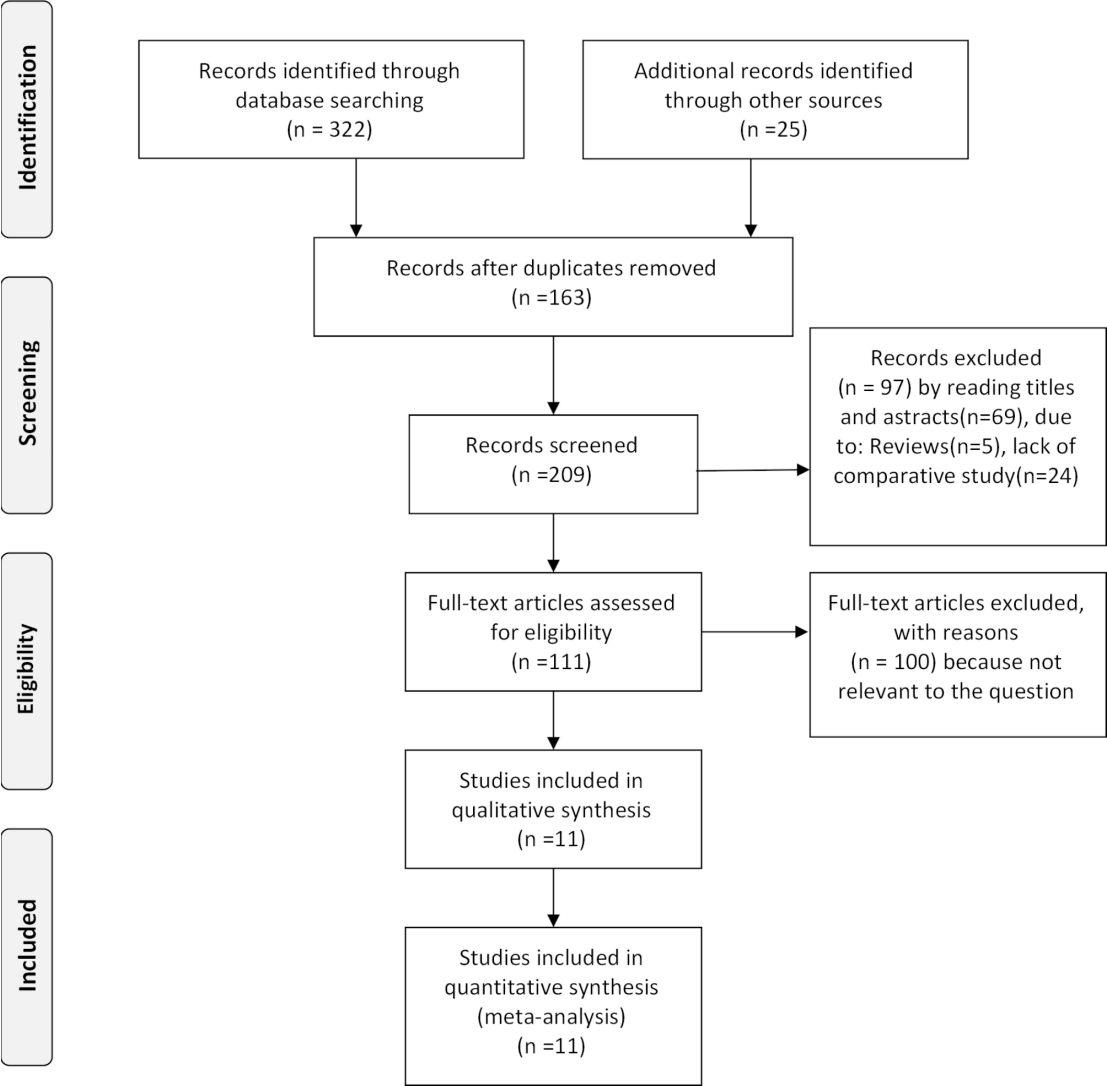
Flow diagram of the published articles evaluated for inclusion in this meta-analysis

As shown in Fig. 1, we identified 347 articles from the electronic search; of these, 163 articles were excluded because of duplication. After screening the titles and abstracts, an additional 97 articles were excluded for various reasons. Finally, 11 publications that met the inclusion criteria were selected for the current meta-analysis [7, 8, 12–20]. A flowchart of the literature search process is shown in Fig. 1.

The characteristics and quality evaluation of the included citations are shown in Table 1. The 11 eligible studied comprised 2 RCTs and 9 retrospective studies. The studies were published from 2012 to 2022 and were performed in Finland, Japan, and the United States. Two studies were published as an abstract and 10 were full-text articles. The participants in the included studies were divided into an MS group (188 participants) and a PS group (308 participants). With respect to NAT, five studies applied NACRT, six studies applied NAC, and one study did not provide the type of NAT. A high risk of bias was not detected in any of the RCTs. According to the Newcastle–Ottawa scale, the study scores ranged from 5 to 7. The characteristics and quality of the included studies are shown in Table 1.

**Table 1:** characteristics of included studies

Author	Country	Design	Type of article	Samples (metal/plastic)	Age, mean (year) (metal/plastic)	Neoadjuvant therapy	Resectable/Boedline resectable	Tumor diameter, mean (mm)	Quality
Adams et al. 2012	USA	Retrospective	Full	9/43	65	NAC	8*44	NA	6
Gardner et al. 2017	USA	RCT	Full	37/26	65.96/65.9	NACRT	Metal 7/10/16 Plastic 3/4/14	33.65/34.2	Moderate
Hasegawa et al. 2021	Japan	Retrospective	Full	27/40	68 /67	NACRT	NA	21/25	6
Kobayashi et al. 2021	Japan	Retrospective	Full	21/22	74/69.5	NACRT	Metal 13/8 Plastic 15/7	20/23	6
Kubota et al. 2014	Japan	Retrospective	Full	17/21	65.9/65.6	NACRT	Metal 0/17 Plastic 0/21	35/35	5
Kuwatani et al. 2020	Japan	Retrospective	Full	17//12	66 /68	NAC	Metal 17/0 Plastic 12/0	21/19	7
Lewis et al. 2018	USA	Retrospective	Abstract	8/5	NA	NAC	NA	NA	7
Nakamura et al. 2018	Japan	Retrospective	Full	17/26	70 /61	NACRT	Metal 8/9 Plastic 17/9	NA	7
Tamura et al. 2021	Japan	RCT	Full	11/11	66.6/67.4	NAC	Metal 0/11 Plastic 0/11	24.6/27.8	Moderate
Tsuboi et al. 2016	Japan	Retrospective	Full	9/11	63/65	NAC	Metal 0/9 Plastic 0/11	25.5/27	6
Vehviläinen et al. 2022	Finland	Retrospective	Full	15/91	64.6/64.8	NA	NA	NA	6

RCT, randomized controlled trials; NAT, neoadjuvant therapy; NAC, neoadjuvant chemotherapy; NRCAT, neoadjuvant chemoradiotherapy; NA, not available

### Outcome measures

#### Reintervention

**Fig. 2:**
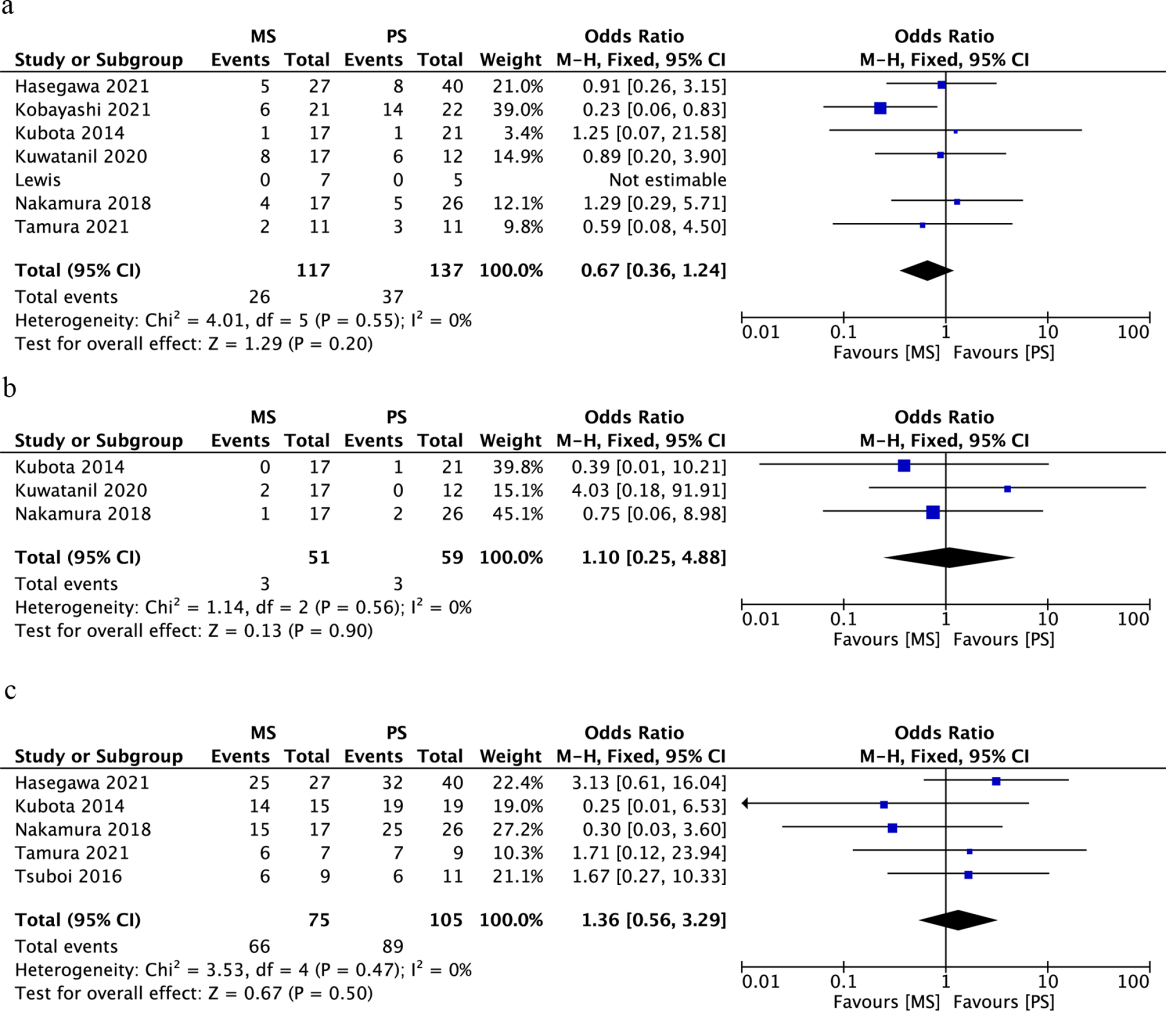
Forest plot of the meta-analysis comparing an MS and PS in terms of (a) reintervention, (b) delay of NAT, (c) RBO, (d) cholangitis, and (e) migration

Four studies involving 333 patients provided data regarding reintervention. The MS group had a lower reintervention rate than the PS group (OR, 0.04; 95% CI, 0.01–0.10; *p* < 0.00001) (Fig. 2a).

#### Delay of NAT

Seven studies provided data regarding delay of NAT. Our meta-analysis showed that patients in the MS group had less delay of NAT than those in the PS group (OR, 0.17; 95% CI, 0.05–0.61; *p* = 0.007) (Fig. 2b).

#### RBO

Nine studies involving 463 patients provided data regarding RBO. The MS group had a lower rate of RBO than the PS group (OR, 0.09; 95% CI, 0.02–0.40; *p* = 0.001) (Fig. 2c).

#### Cholangitis

Four studies involving 218 participants provided data regarding cholangitis after preoperative endoscopic retrograde cholangiopancreatography. Our meta-analysis showed a lower rate of cholangitis in the MS than PS group (OR, 0.16; 95% CI, 0.03–0.79; *p* = 0.03) (Fig. 2d).

#### Migration

There was no significant difference in stent migration between the MS group and PS group (OR, 0.52; 95% CI, 0.15–1.81; *p* = 0.31) (Fig. 2e).

#### Postoperative complications

**Fig. 3:**
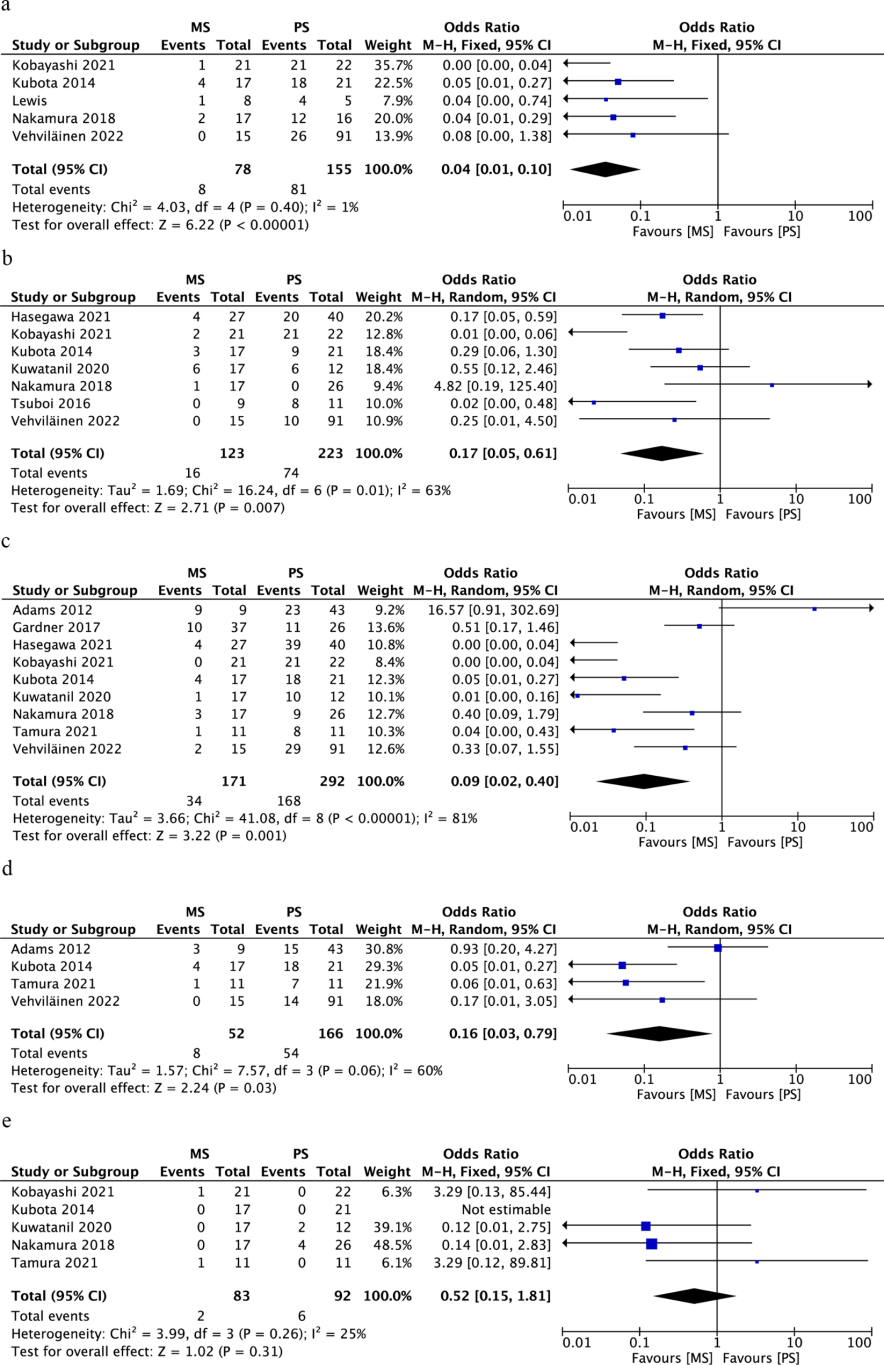
Forest plot of the meta-analysis comparing an MS and PS in terms of (a) postoperative complications, (b) leakage, and (c) R0 resection

Seven studies provided data regarding postoperative complications, and our meta-analysis showed no significant difference between the MS group and PS group (OR, 0.64; 95% CI, 0.36–1.24; *p* = 0.20) (Fig. 3a). The rate of leakage was also comparable between the two groups (OR, 1.10; 95% CI, 0.25–4.88; *p* = 0.90) (Fig. 3b).

#### R0 resection

Five studies involving 180 patients provided data regarding R0 resection. Our analysis showed no significant difference between the two groups (OR, 1.36; 95% CI, 0.56–3.29; *p* = 0.50) (Fig. 3c).

### Sensitivity analysis

The influence of a single study on the overall meta-analysis estimate was investigated by omitting one study at a time. The omission of any study resulted in no significant difference, indicating that our results were statistically reliable.

### Publication bias

The graphical funnel plots of most parameters were symmetrical. Egger’s test revealed no significant publication bias.

## Discussion

The current meta-analysis compared the use of an MS and PS for PBD in terms of preoperative and postoperative outcomes in patients with pancreatic cancer undergoing NAT. To the best of our knowledge, this is the first meta-analysis to focus on this topic. Our meta-analysis showed that compared with a PS, use of an MS for PBD in patients with pancreatic cancer undergoing NAT followed by surgery was associated with lower rates of reintervention, delay of NAT, RBO, and cholangitis. Additionally, the postoperative outcomes were comparable between an MS and PS. Further studies on this topic are recommended.

Because NAT has only recently gained popularity, there are few reports comparing different types of stents during treatment. In patients with pancreatic cancer undergoing NAT, RBO is often the main reason for delay or interruption of NAT. Our study showed that an MS was associated with a lower rate of RBO than a PS (19.88% vs. 57.53%, respectively). Previous studies have shown that the incidence of RBO with use of an MS ranged from 3 to 35%, whereas that with use of a PS ranged from 20–97% [12, 20–22]. The differences in the incidence of RBO among studies may be related to differences in the timing of preoperative NAT. There is no consensus on the optimal time of NAT. The main causes of RBO are stent occlusion and migration. In our study, occlusion occurred less frequently in the MS than PS group. In theory, an MS has a larger diameter than a PS, which can reduce the risk of stent occlusion. Ikezawa et al. [23] demonstrated that a larger-diameter PS may decrease the risk of delayed NAC. Stent migration also plays an important role in occlusion. In the current meta-analysis, the incidence of migration was similar between the MS and PS groups. Consistent with previous studies, our study indicated that reintervention was less frequent with MS than PS placement. This may be related to the fact that MS can reduce the occurrence of RBO. Notably, different MS types may have different impacts on the incidence of stent reintervention. Leone et al. [24] demonstrated that a covered self-expanding MS has longer patency than an uncovered MS. The types of MS varied among the studies included in our meta-analysis, which is one of the shortcomings of our study.

The aim of NAT is to increase the rate of resection and radical surgery. Some studies have shown that MS may be associated with a higher rate of R0 resection because of fewer complications following endoscopic retrograde cholangiopancreatography [16, 25]. Fewer complications may be helpful for enough anticancer agent. However, this conclusion is still debatable. The use of an MS may increase biliary inflammation and fibrosis (adhesion to the bile duct and vessels) [26, 27]. Our study showed no significant difference in R0 resection or postoperative complications between the two groups. Several studies have revealed that an MS was associated with a higher incidence of wound infection and a longer operation time [28, 29]. Tol et al. [30] demonstrated more frequent serious complications with use of a PS than MS. However, the data on the association between an MS and PS on the postoperative complications were limited. Consistent with several previous studies, our meta-analysis showed that an MS did not provide an advantage with respect to postoperative complications. Studies focusing on the effects of an MS and PS on postoperative complications and the R0 resection rate in patients with pancreatic cancer are still limited, and further research on this topic is required.

With respect to cost-effectiveness, an MS appears to have higher cost than a PS; however, this remains controversial. An RCT conducted by Gardner et al. [20] showed that a PS was less expensive than an MS. However, other studies have produced different conclusions. Because costs were calculated differently among previous studies, we were unable to analyze cost-effectiveness in our meta-analysis.

The present study has several limitations. First, most of the included studies were retrospective in nature; only two RCTs were included, which may have led to selection bias. Second, the samples in the included studies were small, and the data provided in the original studies were insufficient. Third, the definitions of reported outcome measures were variable. Finally, the NAT regimens were different, which may have affected the R0 resection rate.

## Conclusion

Use of an MS for PBD in patients with pancreatic cancer undergoing NAT followed by surgery was associated with lower rates of reintervention, delay of NAT, RBO, and cholangitis compared with use of a PS. However, the postoperative outcomes were comparable between the two types of stents. Further studies on this topic are recommended.

## Electronic supplementary material

Below is the link to the electronic supplementary material.

Supplementary Material 1

## Data Availability

All the data used in the study can be obtained from the original articles.

## Abbreviations

NAT: Neoadjuvant therapy
NAC: Neoadjuvant chemotherapy
NACRT: Neoadjuvant chemoradiotherapy
MS: Metal stent
PS: Plastic stent
PBD: Preoperative biliary drainage
CI: Confidence interval
OR: Odds ratios
